# Nanoliposomes from Agro-Resources as Promising Delivery Systems for Chondrocytes

**DOI:** 10.3390/ijms21103436

**Published:** 2020-05-13

**Authors:** Arnaud Bianchi, Émilie Velot, Hervé Kempf, Kamil Elkhoury, Laura Sanchez-Gonzalez, Michel Linder, Cyril Kahn, Elmira Arab-Tehrany

**Affiliations:** 1 Faculté de Médecine, Biopôle de l’Université de Lorraine, Campus Brabois-Santé, Laboratoire UMR 7365 CNRS-Université de Lorraine, Ingénierie Moléculaire et Physiopathologie Articulaire (IMoPA), Université de Lorraine, F-54505 Vandœuvre-Lès-Nancy, France; emilie.velot@univ-lorraine.fr (É.V.); herve.kempf@inserm.fr (H.K.); 2 Laboratoire d’ingénierie des Biomolécules, Université de Lorraine, F-54505 Vandœuvre-Lès-Nancy, France; kamil.elkhoury@univ-lorraine.fr (K.E.); laura.sanchez-gonzalez@univ-lorraine.fr (L.S.-G.); michel.linder@univ-lorraine.fr (M.L.); cyril.kahn@univ-lorraine.fr (C.K.); 3 Campus Brabois-Santé, Laboratoire de Travaux Pratiques de Physiologie, Faculté de pharmacie, Université de Lorraine, F-54505 Vandœuvre-Lès-Nancy, France

**Keywords:** liposomes, chondrocyte, PUFA, drug delivery system, joint regenerative medicine

## Abstract

Investigations in cartilage biology have been hampered by the limited capacity of chondrocytes, especially in rats and humans, to be efficiently transfected. Liposomes are a promising delivery system due to their lipid bilayer structure similar to a biological membrane. Here we used natural rapeseed lecithin, which contains a high level of mono- and poly-unsaturated fatty acids, to evaluate the cytocompatibility of these phospholipids as future potential carriers of biomolecules in joint regenerative medicine. Results show that appropriate concentrations of nanoliposome rapeseed lecithin under 500 µg/mL were safe for chondrocytes and did not induce any alterations of their phenotype. Altogether, these results sustain that they could represent a novel natural carrier to deliver active substances into cartilage cells.

## 1. Introduction

Articular cartilage is an avascular and aneuronal connective tissue nourished by the imbibition from synovial fluid or the blood supplied to the subchondral bone which immediately abuts the cartilage of the joint. Articular cartilage is responsible for distributing the mechanical load and providing the articular joints with a wear resistant surface [1,2]. Biomechanical properties of cartilage are supported by specialized extracellular matrix (ECM). Type II Collagen (*Col II*), aggrecan (*ACAN*), and *Sox-9* (SRY (Sex-Determining Region Y)-Box 9) are genes that codes for ECM molecules specific for cartilage or their regulators [3]. Cartilage is a tissue made of very specific cells called chondrocytes. When chondrocytes belong to joints and thus to articular cartilage, they differentiate and express in their mature functional form an articular identity named articular phenotype. Articular chondrocyte phenotype is primarily identified by the expression of these genes, that are also involved in the maintenance of cartilage anabolism [4,5,6]. ECM composition highly influences the chondrocytes phenotype, since ECM composition and structure changes result in chondrocyte proliferation and loss of the articular phenotype [7,8]. Indeed, changes like proteoglycan content depletion or supramolecular collagen network destabilization can lead to modification of the articular cell phenotype.

In the field of osteoarticular research, one of the main limitations is the very low transfection efficiency of the cells of articular origin by non-viral methods [9,10]. This singularity is particularly marked for human chondrocytes since transfection efficiency yields fluctuating from 1.5% to 35% have been reported with protocols using cationic lipids or cationic polymers derived from polyethyleneimine [9,10,11,12]. The low transfection effectiveness also appears to be typical of the human chondrocytes since transfection above 40% has been reported with non-cationic lipids in bovine or rabbit chondrocytes [11,13,14,15]. This explains why studies requiring a prolonged expression of a transgene in chondrocytes increasingly involve infections with retroviruses, adenoviruses, adeno-associated viruses, or more recently lentiviruses [16,17,18,19,20,21].

Another option to transfect chondrocytes is the use of electroporation, in particular, the Nucleofector^TM^ technology developed by Amaxa Biosystems. This technology achieves transfection efficiencies from 50% up to 70% in primary cultures of human chondrocytes thanks to the association of a commercial salt solution and electrical pulses [10,22,23]. However, the electric pulse used in these methods put cells under high stress (Table S1). Ultimately, a further alternative is to facsimile the nature with liposomes, which are majorly composed of amphiphilic phospholipids. Indeed, liposomes are a promising delivery system due to their lipid bilayer structure similar to a biological membrane, which can safely deliver drugs and maintain their therapeutic levels over extended periods [24]. Moreover, bioactive agents can be protected by liposomes from digestion in the harsh stomach environment, thus enhancing their bioactivity and bioavailability [25,26,27]. Additionally, the composition of the lipids used for nanoliposome bilayers is of interest for cell and tissue response. In this study, natural rapeseed lecithin was used, which have a high level of mono and poly-unsaturated fatty acids mainly linoleic acids (ω -6) and linolenic acids (ω -3) that cannot be produced by the human body and are essential for its health [28,29,30].

Here, in order to evaluate their potential as future carriers of biomolecules for joint regenerative medicine, the cytocompatibility of these rapeseed phospholipids on cultured chondrocytes was tested. Results showed that nanoliposomes were not cytotoxic and induced no death in chondrocytes. In addition, nanoliposomes did not induce changes in the phenotype of chondrocytes. Presented data suggest therefore that nanoliposomes from agro-resources could be very promising candidates to carry active biomolecules into the chondrocytes.

## 2. Results and Discussion

### 2.1. Lipid Classes and Fatty Acid Analysis

A thin-layer chromatography (Iatroscan) was used to separate the lipid classes of lecithin. The major class of rapeseed lecithin phospholipids was phosphatidylcholine (33% ± 0.1%). Rapeseed lecithin triacylglycerols (TAG) percentage was 37.7% ± 0.1% and the polar fraction percentage was 62.3% ± 0.8%.

Monounsaturated fatty acids family was the most abundant fatty acids family in rapeseed lecithin, with C18:1 n-9 (56.51%) present in the highest percentage in this family. The main proportions of fatty acids in the polyunsaturated fatty acids family were C18:2 n-6 (26.32%) and C18:3 n-3 (6.60%), and C16:0 (7.41%) in the saturated fatty acids family.

### 2.2. Nanoliposome Physicochemical Properties

For liposomal systems, physicochemical parameters, such as particle hydrodynamic diameter, size distribution, and electrophoretic mobility values, have to be modulated according to the proposed application [31]. The measured rapeseed nanoliposomes average diameter was 133.1 ± 0.8 nm. The minimum achievable size depends on the material’s viscosity, as well as on the method of preparation parameters.

Particle size distribution is quantified using the dimensionless measure polydispersity index (PDI) [32]. Rapeseed nanoliposomes PDI was 0.19 ± 0.01. Since the PDI value is <0.3, the sample is considered as presenting a narrow distribution [33].

Rapeseed nanoliposomes electrophoretic mobility was −3.41 ± 0.05 μm.cm/Vs. This negative electrophoretic mobility was most probably caused by the presence of different types of anionic phospholipids fractions like phosphatidylglycerol (PG), phosphatidylserine (PS), phosphatidylinositol (PI), and phosphatidic acid (PA) that are negatively charged at physiological pH [34].

**Figure 1:**
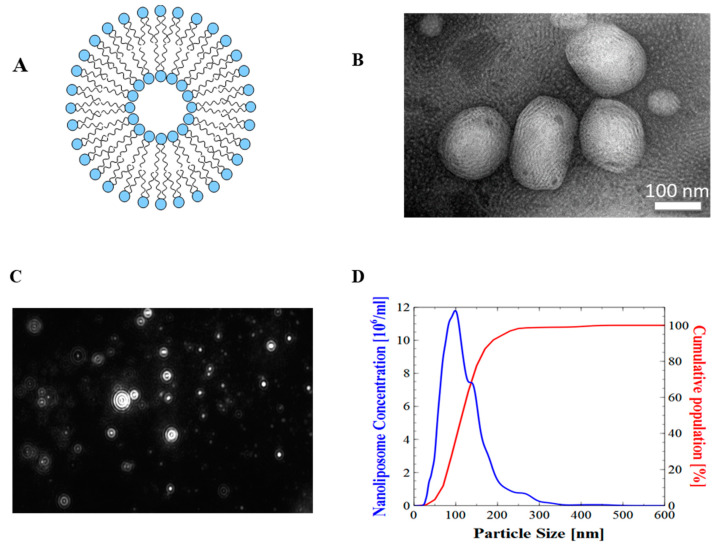
Physical evaluation of nanoliposomes. (**A**) Schematic representation of a nanoliposome; (**B**) transmission electron microscopic images of nanoliposomes; (**C**) nanoliposomes size distribution obtained from NTA (100–400 nm); (**D**) particle hydrodynamic diameter obtained from Nanoparticle Tracking Analysis (NTA) software.

No significant variation was found in nanoliposomes particle hydrodynamic diameter stored at 37 °C between day 0 and day 30. The electrophoretic mobility value remained negative throughout the storage period. The mean particle diameter measured by DLS was similar to the values obtained by Nanoparticle Tracking Analysis (NTA) (Figure 1).

### 2.3. Nanoliposomes Morphology

Surface morphological studies on the shape of the rapeseed liposomes using transmission electron microscopy (TEM) indicated that the systems were almost spherical. The size of rapeseed liposomes produced by sonication and high-pressure homogenization was further proved by the presented TEM images (Figure 1). Minute droplets caused by the presence of weak oil quantity (10%) were observed.

### 2.4. Membrane Fluidity

Lipid membrane fluidity reflects nanoliposomes permeation and the phospholipid alkyl chains order and dynamics in the bilayer [35]. The membrane fluidity of rapeseed nanoliposome was 3.53 ± 0.07. At body temperature, membrane fluidity is affected by the unsaturated fatty acids content [36]. Saturated fatty acids, like stearic acid and palmitic acid, possess straight hydrophobic tails, that interact with one another through van der Waals interactions, and thus favor a more rigid and organized membrane. On the contrary, unsaturated fatty acids, like oleic acid and linoleic acid, possess at least one cis double bond which prevents tight packing, by distorting the hydrophobic chain, and decrease lipid packing and improve membrane fluidity [37,38].

### 2.5. Biocompatibility of Nanoliposomes

**Figure 2:**
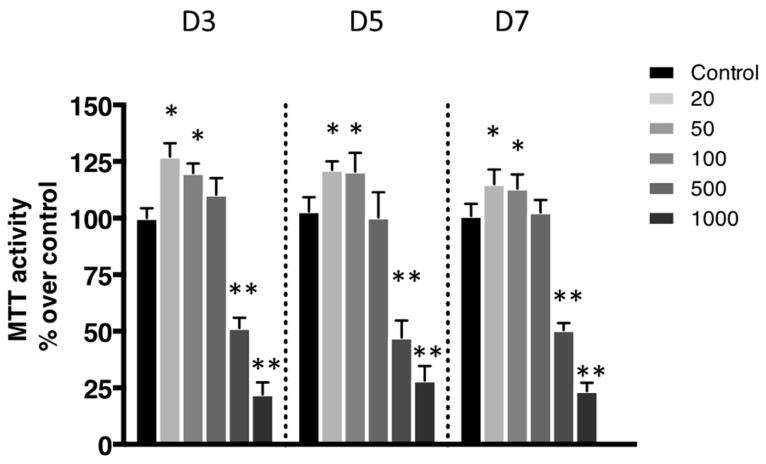
Impact of nanoliposomes on chondrocytes viability. Rat chondrocytes were exposed to increasing concentrations of nanoliposomes (20, 50, 100, 500, and 1000 µg/mL) for 3, 5, and 7 days. Metabolic activity was assessed using the 3-(4,5-Dimethylthiazol-2-yl)-2,5-diphenyltetrazolium bromide (MTT) assay. The cell metabolic activity results on the different membranes are presented in % versus control results (as 100%). The results shown are mean ± SD of at least four individual experiments. * *p* < 0.01 and ** *p* < 0.001 compared to control for each time point.

The metabolic activity was evaluated using a 3-(4,5-Dimethylthiazol-2-yl)-2,5-diphenyltetrazolium bromide (MTT) assay. This metabolic activity was assessed based on the reduction ability of living cells of MTT’s tetrazolium salt into formazan crystals. Most conditions showed significant metabolic activity (Figure 2). The only exceptions were the ones exposed to the most elevated concentrations of nanoliposomes (500 and 1000 µg/mL).

**Figure 3:**
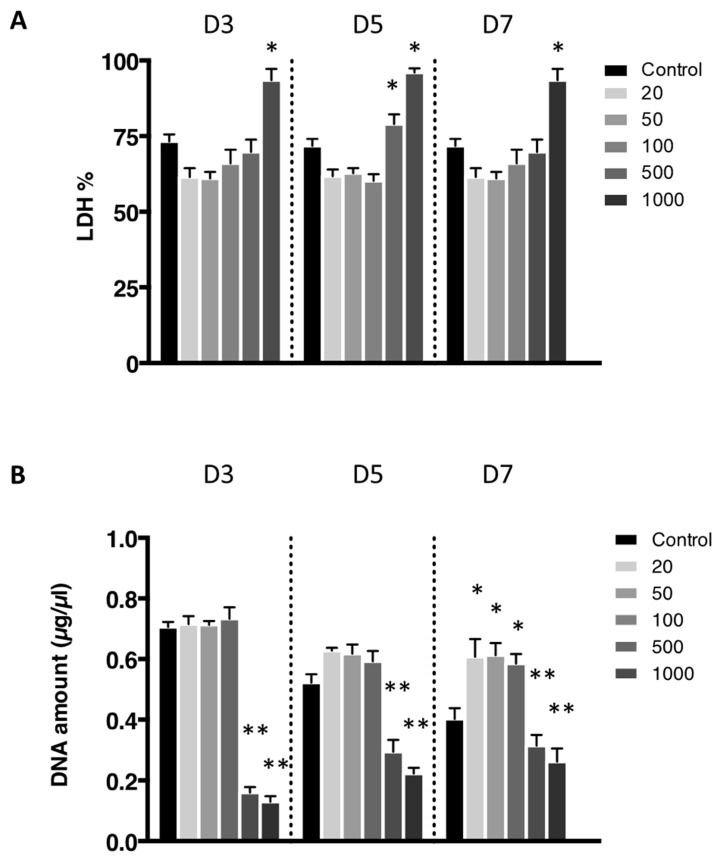
Lactate dehydrogenase activity of chondrocytes in contact with nanoliposomes. Rat chondrocytes were exposed to increasing concentrations of nanoliposomes (20, 50, 100, 500, and 1000 µg/mL) for 3, 5, and 7 days. (**A**) Lactate dehydrogenase (LDH) release was determined as described in Section 3; (**B**) DNA concentrations were measured to estimate cell proliferation. The results shown are mean ± SD of at least four individual experiments. * *p* < 0.01 and ** *p* < 0.001 compared to control for each time point.

The cytotoxicity of the nanoliposomes was evaluated after 3, 5, and 7 days after exposure to various concentrations on free nanoliposomes by lactate dehydrogenase (LDH) assay (Figure 3A). Cells not exposed to nanoliposomes were considered as control. There was no statistically significant cytotoxicity difference between control and the following concentrations of nanoliposomes: 20, 50, 100, and 500 µg/mL. Among the different concentrations, only the highest one, 1000 µg/mL, appeared to be cytotoxic for rat chondrocytes.

**Figure 4:**
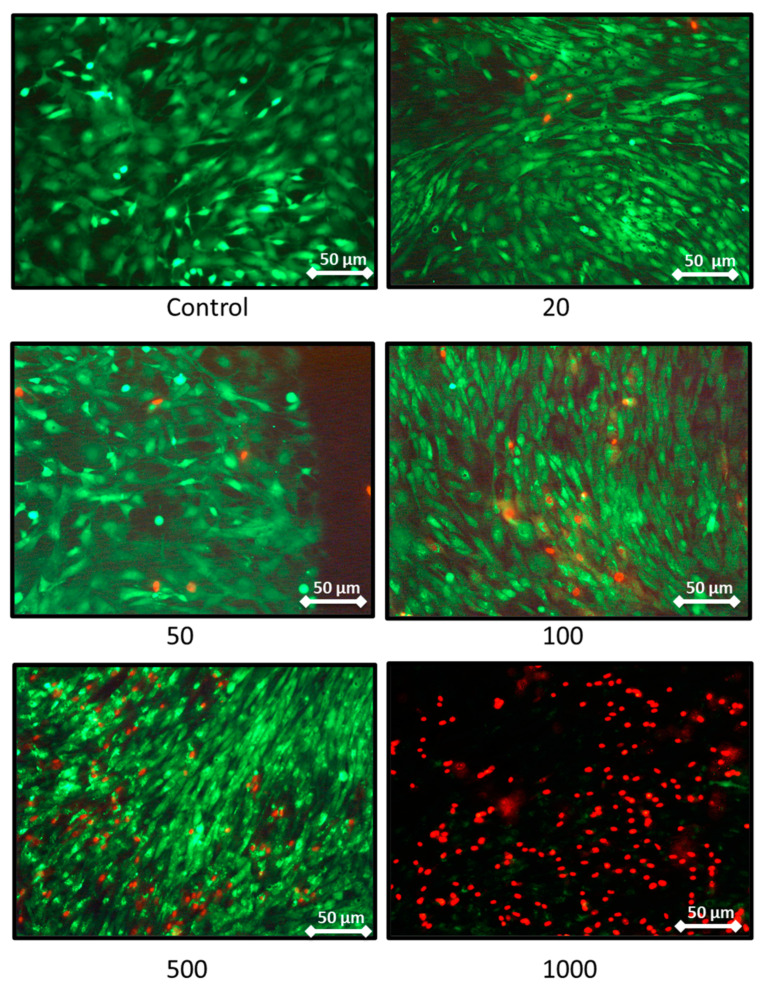
Representative pictures showing the effect of nanoliposome exposure on chondrocytes death. Rat chondrocytes were exposed to increasing concentrations of nanoliposomes (20, 50, 100, 500, and 1000 µg/mL) for 7 days. Live/Dead Cellular Viability/Cytotoxicity Kit from Sigma was used to differentiate live from dead cells. The experiment was performed four times with three biological replicates for each condition tested. Live cells are stained in green and dead cells in red. Immunofluorescence labeling was detected using fluorescence microscopy (DMI 3000B, Leica, Wetzlar, Germany) at 40×, exposure time was between 1 and 5 s. Scale bars on images represents 50 μm.

The effect on the rat chondrocytes proliferation were consistent with the previous results as these two extreme concentrations trigger a decrease in proliferation assessed by DNA quantification (Figure 3B). Moreover, after 7 days of nanoliposome exposure, cell death rises with the increase of nanoliposome concentration as shown in the representative pictures of Figure 4. There is a majority of live cells stained in green for the nanoliposome concentrations of 20, 50, and 100 µg/mL, whereas dead cells stained in red started to spread at 500 µg/mL. A large amount of cell death can be observed following an exposition with the highest concentration of nanoliposomes (1000 µg/mL) (Figure 4).

According to the data illustrated in Figure 3, the concentration of 500 μg/mL significantly reduced cell proliferation (Figure 3B) and did not affect cytotoxicity except slightly at day 5 (Figure 3A) while decreasing metabolic activity (Figure 2), whereas it had a mild effect on cell death (Figure 4). In the extreme, 1000 µg/mL of nanoliposomes induced a decrease in metabolic activity and proliferation and an increase of cytotoxicity leading to chondrocyte death. Those latter observations excluded the 1000 µg/mL from our following experiments.

### 2.6. Effects on Chondrocyte Phenotype

To check for a harmless effect of nanoliposomes on chondrocyte function, RT-PCR analyses were made on expression of specific genes of well-differentiated healthy articular chondrocyte by measuring expression of type II collagen, *ACAN*, and *Sox-9*, known to be decreased when pathological chondrocytes are dedifferentiated towards a fibroblastic or a hypertrophic phenotype. Fibroblastic and/or hypertrophic dedifferentiation is observed in chondrocytes from patients with osteoarthritis (OA).

**Figure 5:**
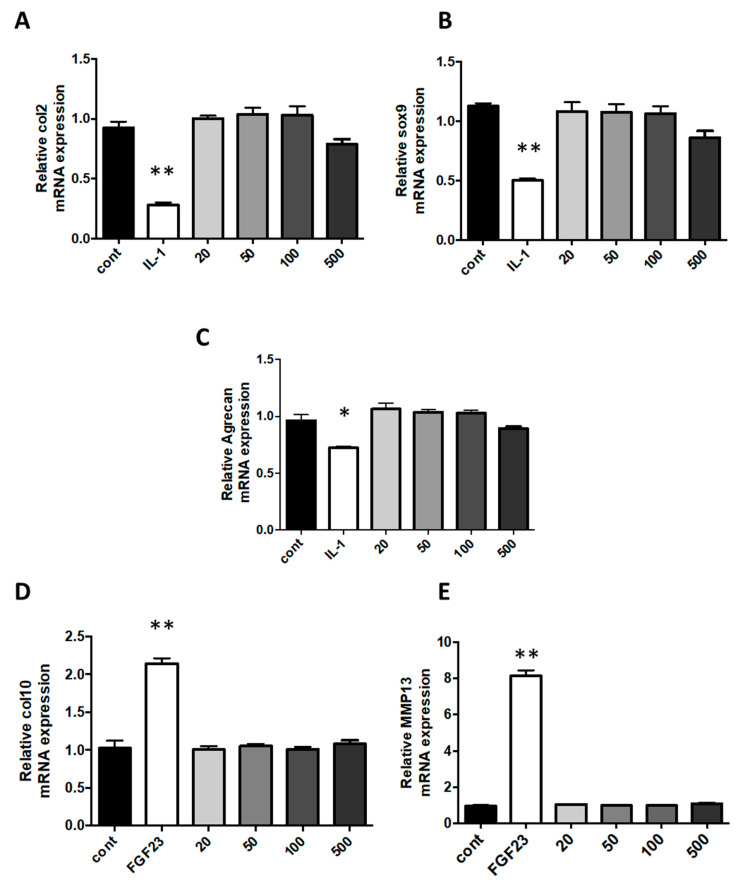
Effect of nanoliposomes exposition on the expression of chondrocytes specific markers. Rat chondrocytes were exposed to increasing concentrations of nanoliposomes (20, 50, 100, and 500 µg/mL) for 24 h or with 10 ng/mL rhIL-1β (A–C) or 100 ng/mL rhFGF23 (D, E). Total RNA was extracted then reverse transcribed into cDNA and analyzed by RT-PCR. The relative abundance of type II collagen (**A**), Sox-9 (**B**), aggrecan (**C**), type X collagen (**D**), and MMP13 (**E**) mRNAs were normalized to RP29 mRNA. The comparison was made by using the ΔΔCt method with the fold value of reference = 1. The results shown are mean ± SD of at least four individual experiments. * *p* < 0.01 and ** *p* < 0.001 compared to control for each time point.

Interleukin (IL)-1β is usually secreted in diseased joints by inflammatory or resident cells. Indeed, it is a major pro-inflammatory cytokine that plays an important role in cartilage degradation and inhibition of ECM synthesis. The stimulation of chondrocytes with IL-1β triggers an undesired fibroblastic dedifferentiation associated with a loss of function [39,40,41]. In rheumatic diseases, occurring cartilage degradation, accompanied by the loss of differentiated phenotype, is significantly affected by the presence of IL-1β. In our work, when cells were challenged with IL-1β, the expression of type II collagen, *Sox-9* and *ACAN* were decreased by 75%, 56%, and 39%, respectively, in comparison to expression in control cells. In cells exposed to various concentrations of nanoparticles, no phenotype modifications were observed at concentrations of nanoliposomes used (Figure 5A–C).

In addition, the hypertrophy switch of the dedifferentiated chondrocyte phenotype has been shown to be another central feature of the osteoarthritis development, which occurs in a similar manner during endochondral ossification with the overexpression of type X collagen and MMP13 (Metalloproteinase 13) mRNA. As we had previously demonstrated that FGF23 was able to induce hypertrophic markers on chondrocytes, we also compared the effect of nanoliposome with FGF23 on hypertrophic dedifferentiation [42]. When cells were challenged with FGF23, in comparison to control cells, expressions of type X collagen and MMP13 were increased 2 and 8-fold, respectively. This was not obtained following nanoliposomes exposition as in these conditions, compared to FGF23, this expression of hypertrophy markers remains very low (Figure 5D,E).

## 3. Materials and Methods

Rapeseed lecithin was acquired from Solae Europe SA society (Le Grand-Saconnex, Switzerland). Methanol and hexane were acquired from Carlo-Erab (France). Boron trifluoride (14% in methanol), acetonitrile (≥99.9%), chloroform (≥99.9%), methanol (≥99.9%) and hexane (≥99.9%) were all purchased from Sigma-Aldrich (Saint-Quentin-Fallavier, France) and Fisher Scientific (Illkirch, France). All the organic solvents were analytical grade reagents. 

### 3.1. Lipid Classes

Lipidic classes of rapeseed lecithin were identified as described previously using an Iatroscan (MK-5 TLC-FID, Iatron Laboratories Inc., Tokyo, Japan) [43]. Each sample was spotted on 10 Chromarod S-III silicacoated quartz rods held in a frame. The rods were developed over 20 min in hexane/diethyl ether/formic acid (80:20:0.2, *v*:*v*:*v*), oven-dried for 1 min at 100 °C, and finally scanned in the Iatroscan analyzer. The Iatroscan was operated under a hydrogen flow rate of 160 mL/min and air flow rate of 2 L/min. A second migration using a polar eluent of chloroform, methanol, and ammoniac (65:35:5, *v*/*v*/*v*) made it possible to quantify polar lipids. The FID results were expressed as the mean value of 10 separate samples. The following standards were used to identify the sample components: Neutral lipids: 1-monostearoyl-rac-glycerol, 1.2-dipalmitoyl-snglycerol, tripalmitin, cholesterol. Phospholipids: l-a-phosphatidylcholine, 3 sn-phosphatidyl-ethanolamine, l-a-phosphatidyl-l-serine, l-a-phosphatidylinositol, lyso-phosphatidylcholine, sphingomyelin. All standards were purchased from Sigma-Aldrich (Saint-Quentin-Fallavier, France). The recording and integration of the peaks were performed using the ChromStar (Weyhe, Germany) internal software.

### 3.2. Fatty Acids Composition

Fatty acid methyl esters (FAMEs) were analyzed as previously described [44]. In brief, FAMEs separation was carried out on a gas chromatography (Perichrom, Saulx-lès-Chartreux, France) equipped with a flame-ionization detector, by setting the injector and detector temperatures at 250 °C and by initially setting the fused silica capillary column temperature at 120 °C for 3 min, increasing it later at a rate of 2 °C.min^−1^ to 180 °C, then adjusting it to 220 °C for 25 min. Polyunsaturated fatty acids standard mixtures (Supelco, Sigma-Aldrich, Bellefonte, PA, USA) were used to categorize fatty acids. All runs were performed in triplicate.

### 3.3. Nanoliposomes Preparation

A total of 49 mL of distilled water was added to 1 g of rapeseed lecithin and agitated under nitrogen for 5 h. Samples were then probe-sonicated at 40 kHz for 5 min (1 s on, 1 s off) in an ice bath, followed by homogenization using a high-pressure homogenizer (EmulsiFlex-C3, Sodexim SA, France). Homogenization was achieved by introducing 50 mL quantities under a pressure of 1500 bar for 7–8 cycles. Produced liposomes were stored in glass bottles in the dark at 4 °C until use.

### 3.4. Nanoliposomes Physicochemical Characterization

Nanoliposomes size, PDI, and electrophoretic mobility were analyzed using a Malvern Zetasizer Nano ZS (Malvern Instruments, Worcestershire, UK) dynamic light scattering (DLS). Following dilution (1:400) with ultra-filtrate distilled water, samples were characterized at a scattering angle of 173 °, a refractive index of 1.471, an absorbance of 0.01, and at a temperature of 25 °C.

### 3.5. Nanoparticle Tracking Analysis (NTA)

Nanoliposomes were examined with a NanoSight LM10 instrument (Salisbury, Wiltshire, UK) as previously described [24]. In brief, the suspension was diluted using a monochromatic laser beam at 405 nm. NTA software was used to analyze particle movement in the 60 s video recorded at a frame rate of 30 frames/s, to identify the mean, mode, and median particle size, in addition to a concentration estimation. All measurements were performed in triplicate at a dilution of 1:10,000.

### 3.6. Nanoliposomes Stability

Nanoliposomes were purged under nitrogen and stored at 37 °C for 30 days in a drying cupboard. All physicochemical parameters were analyzed every 3 days of the 30 days storage duration.

### 3.7. Nanoliposomes Transmission Electron Microscopy (TEM)

To monitor the nanoliposomes microstructure, TEM was employed using a negative staining method as described previously [45]. Briefly, nanoliposomes concentration was reduced by a 30-fold dilution with distilled water. A drop of a solution of 2% ammonium molybdate and the diluted samples was placed for 5 min on a Formvar-carbon coated copper grid (200 mesh, 3 mm diameter HF 36). The mesh was observed using a Philips CM20 TEM.

### 3.8. Membrane Fluidity

Nanoliposomes membrane fluidity was determined by measuring the fluorescent intensity of TMA–DPH according to the conventional method [35]. In brief, TMA–DPH (in ethanol) solution was added to the nanoliposome suspension and incubated for more than 1 h at ambient conditions while stirring gently. The mixture was distributed into 96-well black microplate (200 μL of solution per well). Samples fluorescence intensity was measured using a Tecan INFINITE 200R PRO (Grödig, Austria). Under constant stirring at 25 °C, the emission of excited samples at 360 nm was registered at 430 nm. The polarization value (*P*) of TMA–DPH was calculated using the following equation:

$$P=\frac{I_{II}-GI_{⊥}}{I_{II}+2GI_{⊥}}$$

where *I_II_* is the fluorescent intensity parallel to the excitation plane, *I*_⊥_ the fluorescent intensity perpendicular to the excitation plane, and *G* is the factor that accounts for transmission efficiency. Membrane fluidity is defined as 1/*P*. The results were measured in triplicate.

### 3.9. Chondrocytes Isolation and Culture

Chondrocytes were isolated and cultured as previously detailed [46]. In brief, they were isolated from Wistar male rats, that were euthanized under general anesthesia according to European animal care guidelines. Sequential digestion using pronase and collagenase was used to obtain cells, that were then washed with PBS and cultured to confluence at 37 °C and 5% CO_2_ in 75 cm^−1^ flasks_._ DMEM/Ham’s F-12 supplemented with streptomycin (100 µg/mL), penicillin (100 U/mL), and L-glutamine (2 mM) was used as the culture medium, with the addition of either 1% fetal calf serum (FCS) during experiments or 10% heat-inactivated (FCS) during subcultures. Chondrocytes were used at passage number 1. The mRNA levels of highly specific cartilage genes (*ACAN, type II collagen*, and *Sox-9*) were used to assess the chondrocytes differentiated articular phenotype.

Chondrocytes maintained in low FCS medium (1%) were incubated in the presence or absence of nanostructures at 20, 50, 100, 500, and 1000 µg/mL for 3, 5, or 7 days.

### 3.10. Biocompatibility Assays

Nanoliposomes impact on cell behavior was investigated using cytotoxicity, cell metabolic activity, and cell proliferation parameters.

#### 3.10.1. Cytotoxicity Evaluation by LDH Assay

The cytotoxicity test was completed with the Cytotoxicity Detection Kit^PLUS^ (LDH) (Mannheim, Germany) following the manufacturer’s instructions. The basis of this assay is the measurement of LDH activity released from damaged cell cytosol. Three controls are involved: high control (maximum LDH release), low control (untreated cells), and background control (assay medium). The absorbance was read on a spectrophotometer at 490 nm (Varioskan^®^ Flash, Thermo Scientific, Waltham, MA, USA). The average absorbance values of the triplicate samples and controls were calculated and subtracted from the background control absorbance values, to determine the experimental absorbance values. The percentage of cytotoxicity was determined over the value of the high control (fixed to 100).

#### 3.10.2. Cell Proliferation

Cell proliferation was assessed after 3, 5, or 7 days of chondrocyte culture using Hoechst assay, which allows cell DNA quantification. Briefly, chondrocytes were harvested from 12-well plates and suspended in 100 µL of Hoechst buffer (0.1 M of NaCl, 1mM EDTA, and 10 mM TRIS, pH 7.4). This was followed by five series of freezing/thawing cycles to rupture cells and release their DNA. Black fat-bottom plates with low fluorescent background were used to perform the assay that was quantified using a calf thymus DNA standard curve. Samples were mixed with 2 µL of Hoechst solution and measurements of DNA samples and standards were performed by fluorescence spectrophotometry. Each sample’s DNA concentration was based on its fluorescence measurement compared to the standard curve.

#### 3.10.3. Cell Metabolic Activity

Cell metabolic activity was measured using MTT ((3-(4,5-dimethylthiazol-2-yl)-2,5-diphenyltetrazolium bromide) assay. A total of 50 µL of MTT solution was added to 200 µL of cell culture medium. Chondrocytes were incubated for 4 h at 37 °C, 5% CO_2_, and 95% humidity. Then, the supernatant was removed, protected from light, dissolved with 200 µL DMSO, lightly mixed for 5 min at 37 °C, centrifuged, and its absorbance was read using a spectrophotometer within 30 min at 540 nm. The control condition for chondrocyte metabolic activity was used as the reference value.

### 3.11. Live/Dead Cell Assay

Live/Dead Cellular Viability/Cytotoxicity Kit from Molecular Probes (Fisher Scientific, Illkirch, France) was used to discriminate live cells from dead cells. The kit is composed of two molecular reporters: Calcein-AM, an acetoxymethyl ester of calcein, highly lipophilic and cell membrane permeable, and propidium iodide (PI). Live cells are stained in green with Calcein-AM, whereas dead cells are stained red with PI. Cellular viability assays were performed 3, 5, or 7 days after exposition to various concentrations of nanoliposomes. The experiment was performed three times with three biological replicates for each condition tested.

### 3.12. RNA Isolation, Reverse Transcription and Real-Time Polymerase Chain Reaction (RT-PCR)

Total RNAs were isolated from cultured chondrocytes, using the Nucleospin RNA kit^®^ (Macherey Nagel, Hoerdt, Germany) according to the manufacturer’s instructions. A total of 200 nanograms of total RNAs were reverse-transcribed at 37 °C for 90 min in a 20 μL reaction mixture containing 200 U Moloney Murine Leukemia Virus reverse transcriptase (Invitrogen, Fisher Scientific, Illkirch, France), 1.5 mM MgCl_2_, 5 μM random hexamer primers, and 10 mM dNTP. A Mastercycler gradient thermocycler (Eppendorf, Hamburg, German) was used to produce cDNAs. Subsequently, RT-PCR was completed using Step One Plus^TM^ (Applied Biosystems, Fisher Scientific, Illkirch, France) technology with specific primers (Table 1) and iTAQ SYBRgreen^TM^ master mix system (Bio-Rad, Steenvoorde, Netherlands).

RT-PCR reagents were added at the concentrations recommended by the manufacturer. The specific PCR products melting temperature was determined using a melting curve. Following amplification, a 1% agarose gel stained with Gel Red (Biotium, Interchim, Montlucon Cedex, France) was used to check the product size. Positive and negative reaction controls were included in each run. The mRNA levels of the gene of interest and of the ribosomal protein 29 (*RP29*) were quantified in parallel for each sample using the ΔΔCt method. Finally, the results were presented as fold expression over the proper control.

### 3.13. Statistical Analysis

Results are expressed as the mean ± SD. Statistical analyses were performed with GraphPad Prism 6 (GraphPad Software, San Diego, CA, USA) using one-way ANOVA multiple comparisons followed by Tukey correction. *p*-values were indicated in the legends if considered significant (* *p* < 0.01, ** *p* < 0.005).

## 4. Conclusions

Except for a high concentration (1000 µg/mL), our results showed that rapeseed lecithin nanoliposomes were safe for chondrocytes viability and integrity. Indeed, in our experimental conditions, we demonstrated here that nanoliposomes (i) do not induce cytotoxicity and cell death of chondrocytes, (ii) do not alter basal type II collagen, *Sox-9,* and *ACAN* expressions, and (iii) do not induce *type X collagen* and *Mmp13* expressions, which are both indicative of a preserved phenotype. Those in vitro experiments strongly suggest that rapeseed lecithin nanoliposomes could be used as natural carriers to deliver active substances into cartilage cells.

In future experiments, nanoliposome rapeseed lecithin needs to be further tested on cartilage explant and in vivo directly into the joint to confirm our in vitro results, and thus validate nanoliposomes as an attractive novel alternative for delivering active substances into cartilage in regenerative medicine.

## Figures and Tables

**Table 1:** Sequences of specific primers for RT-PCR analyses.

Genes	Sequences 5′-3′
ACAN	Fw: CAA-CCT-CCT-GGG-TGT-AAG-GA
	Rev: TGT-AGC-AGA-TGG-CGT-CGT-AG
Sox-9	Fw: CTG-AAG-AAG-GAG-AGC-GAG-GA
	Rev: GGT-CCA-GTC-ATA-GCC-CTT-CA
Col II	Fw: TCC-CTC-TGG-TTC-TGA-TGG-TC
	Rev: CTC-TGT-CTC-CAG-ATG-CAC-CA
Col X	Fw: ATA-TCC-TGG-GGA-TCC-AGG-TC
	Rev: TGG-GTC-ACC-CTT-AGA-TCC-AG
MMP13	Fw: CTT-CTG-GCA-CAC-GCT-TTT-CC
	Rev: AGC-TGC-TTG-TCC-AGG-TTT-CA
RP29	Fw: CTC-TAA-CCG-CCA-CGG-TCT-GA
	Rev: ACT-AGC-ATG-ATT-GGT-ATC-AC

ACAN: aggrecan; Sox-9: SRY (Sex-Determining Region Y)-Box 9; Col II: type II collagen; Col X: type X collagen; MMP: metalloproteinase; RP: ribosomal protein.

## Supplementary Materials

Supplementary materials can be found at https://www.mdpi.com/1422-0067/21/10/3436/s1. Table S1: Impact of electroporation on chondrocyte survivability.
