# Supplementary material for: Nanoliposomes from Agro-Resources as Promising Delivery Systems for Chondrocytes
Source: Int J Mol Sci. 2020 May 13;21(10):3436. doi: 10.3390/ijms21103436 (PMC7279141; doi:10.3390/ijms21103436)
Supplement: Supplementary file 1 [file ijms-21-03436-s001.pdf]

International Journal of
Molecular Sciences

MDPI

Table S1. Impact of electroporation on chondrocyte survivability.

Conditions	1% FCS	10% FCS	15% FCS
No Plasmid	100 ± 3	100 ± 4	100 ± 2
500 ng	50 ± 9 *	65 ± 7 *	70 ± 5 **
1 µg	48 ± 8 *	51 ± 11 *	62 ± 7 *
2 µg	40 ± 5 **	48 ± 7 *	61 ± 8 *

Rat chondrocytes were electroporated with plasmids encoding pCMV Renilla luciferase before electroporation using a Nucleofector kit (Amaxa/Lonza) according to the manufacturer’s protocol. Briefly, 3 × 106 cells were gently mixed with 0.5, 1, or 2 µg of plasmid and immediately after transfection, cells were split equally into three wells containing 1%, 10%, or 15% fetal calf serum (FCS)-DMEM/Ham’s F-12 supplemented with L-glutamine (2 mM), penicillin (100 U/mL), streptomycin (100 µg/mL), left to recover for 24 h. Metabolic activity was assessed using the MTT assay. The cell metabolic activity results are presented in % versus control results (as 100%). The results shown are mean ± SD of at least four individual experiments. * $p < 0.01$ and ** $p < 0.001$ compared to control for each % FCS with no plasmid.
